# Lipid production through simultaneous utilization of glucose, xylose, and l-arabinose by *Pseudozyma hubeiensis*: a comparative screening study

**DOI:** 10.1186/s13568-016-0236-6

**Published:** 2016-08-26

**Authors:** Ayumi Tanimura, Masako Takashima, Takashi Sugita, Rikiya Endoh, Moriya Ohkuma, Shigenobu Kishino, Jun Ogawa, Jun Shima

**Affiliations:** 1 Division of Applied Life Sciences, Graduate School of Agriculture, Kyoto University, Kitashirakawa Oiwake-cho, Sakyo-ku, Kyoto, 606-8502 Japan; 2 Japan Collection of Microorganisms, RIKEN BioResource Center, Koyadai, Tsukuba, Ibaraki 305-0074 Japan; 3 Department of Microbiology, Meiji Pharmaceutical University, Noshio, Kiyose, Tokyo 204-8588 Japan; 4 Faculty of Agriculture, Ryukoku University, Seta Oe-cho, Otsu, Shiga 520-2194 Japan

**Keywords:** Oleaginous yeast, Fatty acids, Xylose, l-arabinose, *Pseudozyma hubeiensis*

## Abstract

Co-fermentation of glucose, xylose and l-arabinose from lignocellulosic biomass by an oleaginous yeast is anticipated as a method for biodiesel production. However, most yeasts ferment glucose first before consuming pentoses, due to glucose repression. This preferential utilization results in delayed fermentation time and lower productivity. Therefore, co-fermentation of lignocellulosic sugars could achieve cost-effective conversion of lignocellulosic biomass to microbial lipid. Comprehensive screening of oleaginous yeasts capable of simultaneously utilizing glucose, xylose, and l-arabinose was performed by measuring the concentration of sugars remaining in the medium and of lipids accumulated in the cells. We found that of 1189 strains tested, 12 had the ability to co-ferment the sugars. The basidiomycete yeast *Pseudozyma hubeiensis* IPM1-10, which had the highest sugars consumption rate of 94.1 %, was selected by culturing in a batch culture with the mixed-sugar medium. The strain showed (1) simultaneous utilization of all three sugars, and (2) high lipid-accumulating ability. This study suggests that *P. hubeiensis* IPM1-10 is a promising candidate for second-generation biodiesel production from hydrolysate of lignocellulosic biomass.

## Introduction

The lipid produced by microorganisms is considered to have powerful potential for the development of a new kind of energy, and has received significant interest from sustainable energy researchers. Lipid accumulated by oleaginous yeast is viewed as a promising alternative to second-generation biodiesel, since the composition of the fatty acids produced by yeast is suitable for biodiesel production. That is, it contains palmitic (16:0), stearic (18:0), oleic (18:1), and linoleic (18:2) acids at a high ratio, mainly in the form of triacylglycerol (TAG) (Beopoulos et al. 2011; Knothe 2009; Meng et al. 2009; Sitepu et al. 2014). Compared to other oleaginous microorganisms, oleaginous yeasts are advantageous due to their rapid growth rate (Li et al. 2008), and they are deemed to have the potential to convert various carbon sources, such as cellobiose, xylose and starch, to lipid (Gong et al. 2012; Hu et al. 2011; Huang et al. 2014; Tanimura et al. 2014a).

Second-generation biodiesel is made from non-food sources such as rice straw, wood residue, corncob, and sugarcane bagasse. Lignocellulosic hydrolysates from these feedstocks are composed mainly of glucose, xylose, and l-arabinose (hereafter referred to simply as arabinose) (Huang et al. 2009; Kumar et al. 2009; Madhavan et al. 2012; Roberto et al. 1995; Tsigie et al. 2011). The ratio of the sugars and their concentration in the hydrolysates vary depending on the feedstock used and pretreatment conditions (Behera et al. 2014; Kumar et al. 2009). A previous study investigated lipid accumulation using a medium containing 3 % glucose by *Vanrija musci* JCM 24512 (formally *Cryptococcus musci*) (Tanimura et al. 2014b). The strain showed higher lipid-producing ability from glucose compared to typical oleaginous yeasts such as *Lipomyces starkeyi* and *Rhodosporidium toruloides*. Strains like this that can convert glucose to lipid with high productivity are well suited for the production of glucose-rich hydrolysate such as the hydrolysate of starchy biomass. However, because pentoses content ranged from 20 to 40 % of the total released sugars (Sumphanwanich et al. 2008; Tanimura et al. 2012), glucose utilization alone is insufficient for the conversion of lignocellulosic biomass. In other words, sequential utilization of the sugars extends fermentation times. Therefore, economically feasible production of lipid will require a yeast strain with the ability to co-ferment the lignocellulosic sugars.

Research has shown that engineered yeast can be valuable in expanding the substrate range. For example, Tai engineered *Yarrowia lipolytica* to make it utilize xylose (Tai 2012). In that case, the xylose reductase encoding gene (*XYL1*) and xylitol dehydrogenase encoding gene (*XYL2*) were transferred from the xylose-fermenting yeast *Scheffersomyces stipitis* into the strain. The uptake of arabinose has not yet been reported, and therefore, research in this area is expected. In addition, to avoid the problem caused by glucose repression, the quest for novel oleaginous yeasts able to co-ferment glucose, xylose, and arabinose would seem to be an efficient strategy. To the best of our knowledge, there has not yet been a screening study of oleaginous yeasts able to ferment the three sugars. The application of the following new oleaginous yeasts to the conversion of lignocellulosic sugars to lipids has been carried out: *Trichosporon fermentans* (Huang et al. 2009, 2014), *L. starkeyi* (Anschau et al. 2014), *Cryptococcus curvatus* (Liang et al. 2014), *R. toruloides* (Wiebe et al. 2012) and *Y. lipolytica* (Tsigie et al. 2011). However, in these sugar-consumption profiles, sequential utilization of arabinose was not observed.

In this study, exhaustive screening of 1189 isolates was undertaken to identify an oleaginous yeast strain that was able to convert the glucose, xylose, and arabinose in artificial hydrolysate to lipid. We here report the discovery of *Pseudozyma hubeiensis* IPM1-10, which shows a significant utilization of a mixture of the sugars.

## Materials and methods

### Strains and media

Yeast strains collected and taxonomically identified by Takashima et al. (2012) were our primary resources. Yeast strains isolated by Dr. Ando, Kyoto University, from the Kushiro and Kyoto area (Japan) were also assessed. YM agar medium (Difco, Detroit, MI, USA) was used for pre-culture and maintenance of yeast strains.

The artificial hydrolysate of lignocellulosic biomass (mixed-sugar medium) was based on the medium used by Gong et al. (2012), which contained ammonium sulfate 1 g/L, yeast extract 0.5 g/L, potassium dihydrogenphosphate 1 g/L, magnesium sulfate 1 g/L, glucose 20 g/L, xylose 10 g/L and arabinose 5 g/L. The single sugar medium contained ammonium sulfate 1 g/L, yeast extract 0.5 g/L, potassium dihydrogenphosphate 1 g/L, magnesium sulfate 1 g/L, and glucose 35 g/L or xylose 35 g/L or arabinose 35 g/L.

### Screening

**Fig. 1:**
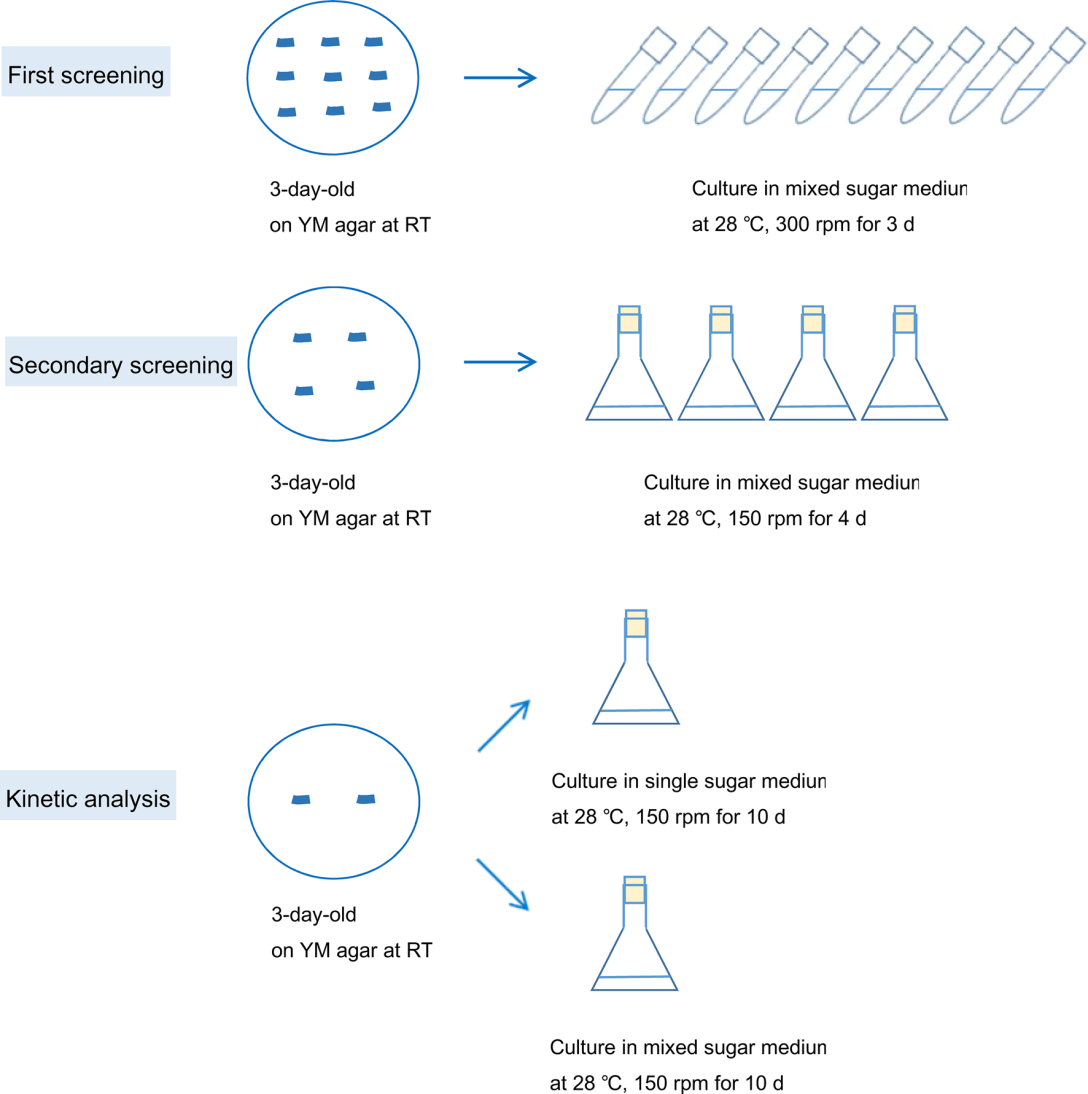
Experimental flow scheme

The screening procedure is depicted in Fig. 1. For the first round of screening, one loop of 3-day-old yeast culture was suspended in 3 mL of mixed-sugar medium in a test tube, and incubated for 3 days at 28 °C, with reciprocal shaking at 300 rpm. Sugar concentrations of the culture supernatants were determined by HPLC, as described below.

In the secondary screening, the yeast strains selected by the first screening were used. These strains have been deposited in the Japan Collection of Microorganisms (JCM). One loop of 3-day-old yeast culture was suspended in 25 mL of mixed-sugar medium in an Erlenmeyer flask and incubated at 28 °C, with rotary shaking at 150 rpm. The process was performed in batch culture. Culture broth was withdrawn after 4 days. Sugars concentrations of the supernatants were determined by HPLC. Cells from culture broth were harvested by centrifugation (15,000 rpm for 10 min), and washed with distilled water. Cell mass was determined by dry weight after lyophilization. Intracellular total lipids were determined by gas chromatography, as described below.

### Kinetic analysis of selected strains

The yeast strains screened by the secondary screening were used. One loop of 3-day-old yeast culture was suspended in 100 mL of mixed-sugar medium and single sugar medium in Erlenmeyer flasks and incubated at 28 °C, with rotary shaking at 150 rpm for 10 days. All of the experiments were performed in batch culture. Fermentation broth was withdrawn at specific time intervals, and intracellular total lipids and sugar concentrations were determined. All experiments were performed in triplicate.

### Measurement of fatty acids

Total intracellular lipid was estimated as total fatty acids. The accumulated lipid of the yeast strain was extracted from the lyophilized cells by a hydrochloric acid-catalyzed direct methylation method (Ichihara and Fukubayashi 2010). In brief, after cultivation, the centrifuged cells were lyophilized and weighed. The cells were suspended in toluene and methanol, then directly transmethylated with 8 % methanolic HCl at 100 °C for 1 h. The resultant fatty acid methyl esters were extracted with n-hexane and analyzed using a gas chromatograph (GC-2010 Plus; Shimadzu, Kyoto, Japan) equipped with a flame ionization detector (FID) and an autosampler (AOC20; Shimadzu). A TC-17 capillary column (GL Science, Tokyo, Japan) was used. Heptadecanoic acid (C17: 0) was used as an internal standard for the determination of fatty acid concentrations.

### Measurements of sugars

Glucose, xylose, and arabinose concentrations were determined using an HPLC (Shimadzu, Kyoto, Japan) equipped with an Aminex Fermentation Monitoring Column (Bio-Rad Laboratories, Hercules, CA, USA) and Micro-Guard Cation H Refill Cartridges with a Standard Cartridge Holder (Bio-Rad Laboratories). The detector was an RID 10A refractive index detector (Shimadzu). The column was kept at 60 °C using a CTO 20A column oven (Shimadzu). Sulfuric acid solution (5 mM) was used as the mobile phase at a constant flow rate of 0.6 mL/min.

## Results

### Screening

As mentioned above, the experimental flow scheme is shown in Fig. 1. A total of 1189 yeast strains were tested in test tubes containing 3 mL of mixed-sugar medium during the first screening step. The consumed glucose, xylose, and arabinose concentration ranged from 0–20 g/L (0–100 %), 0–5.8 g/L (0–58 %) and 0–5 g/L (0–100 %), respectively. Twelve oleaginous yeast strains with relatively high sugar-consuming ability were obtained through the process (Table 2). Among the 12 yeast strains selected, seven strains belonged to *P. hubeiensis*.

**Fig. 2:**
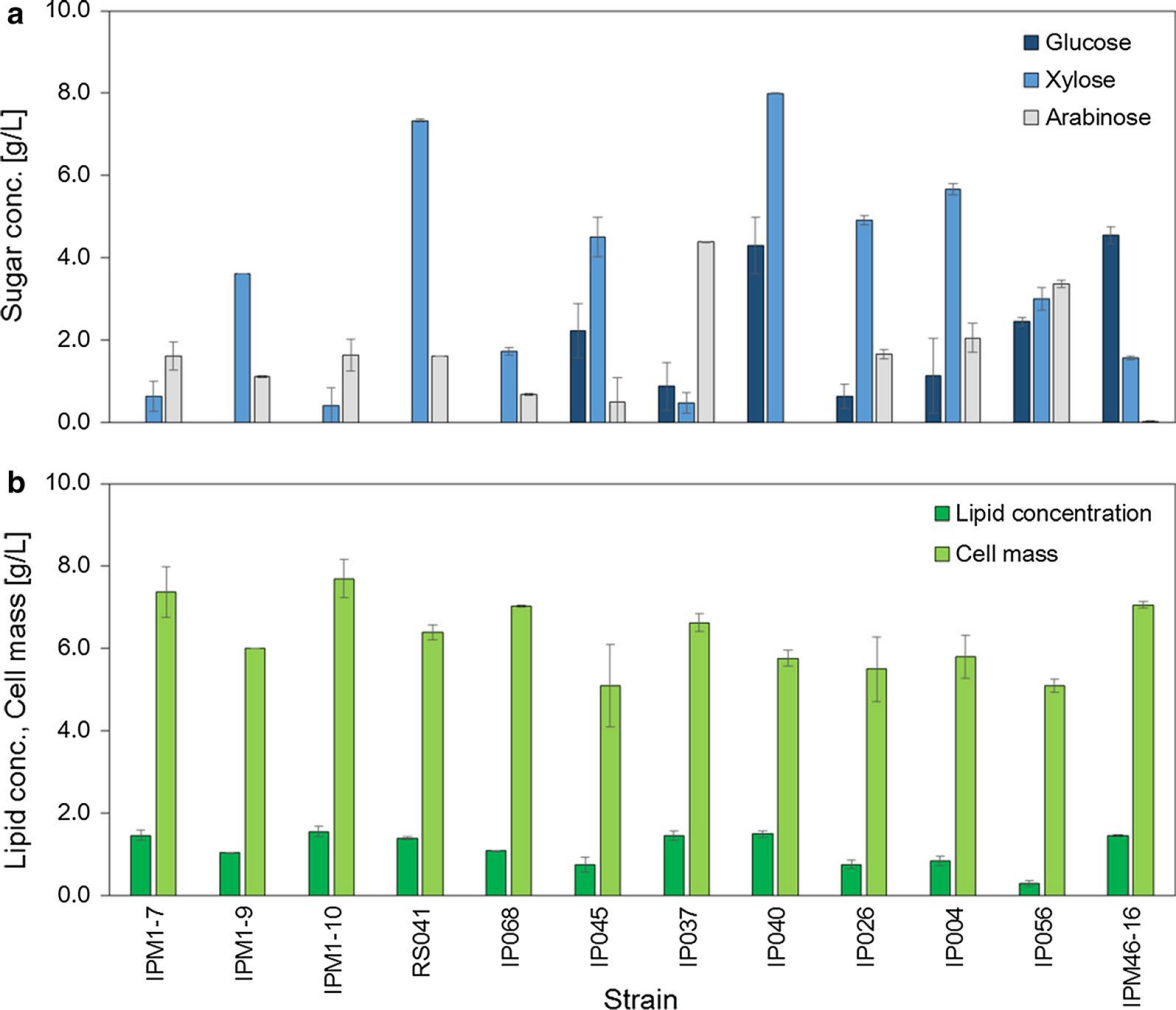
Residual sugars (**a**) and lipid concentration (**b**) of 12 selected oleaginous yeast strains after a 4-day culture. Data are mean ± standard deviation (*error bars*) of three assays

In the secondary screening grown in 25 mL of mixed-sugar medium in batch culture, sugars and lipid concentration were measured after 4 days of fermentation (Fig. 2). All the tested yeast strains showed pentose-assimilating ability. *P. hubeiensis* IPM1-10 consumed 94.1 % of total sugar (Fig. 2a). The strain produced approximately 1.56 g/L, which was higher than the lipid concentrations (Fig. 2b) of the other selected strains. Therefore, this strain was selected for further studies.

### Sugar consumption and lipid production by *P. hubeiensis* IPM1-10

**Fig. 3:**
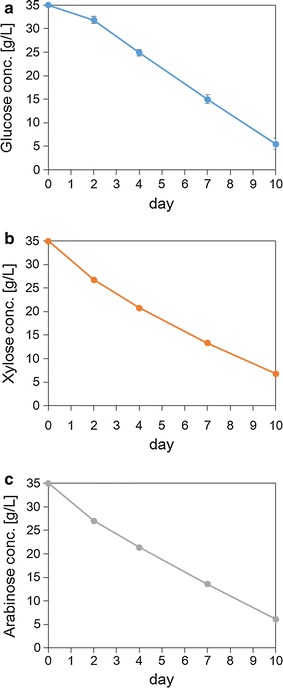
Time course analyses of *P. hubeiensis* IPM1-10: consumption of glucose (**a**), xylose (**b**), and arabinose (**c**) in single-sugar medium. Data are mean ± standard deviation (*error bars*) of three assays

**Fig. 4:**
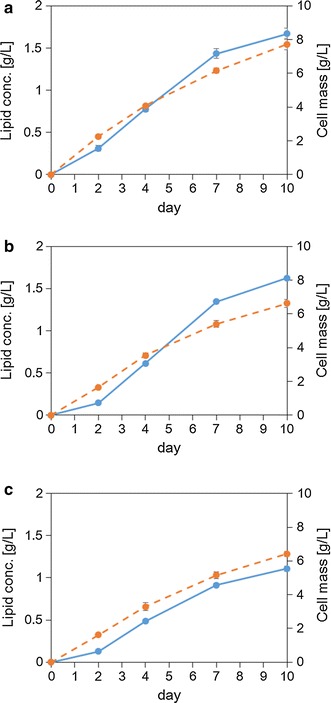
Time-course analyses of *P. hubeiensis* IPM1-10: lipid concentration (*solid line*) and in cell mass (*broken line*) in the medium containing glucose (**a**), xylose (**b**), and arabinose (**c**) as a carbon source. Data are mean ± standard deviation (*error bars*) of three assays

To investigate the selected strain, *P. hubeiensis* IPM1-10, the lipid-accumulating ability and sugar-consumption profile were determined using three kinds of single-sugar medium in batch culture. The medium contained 35 g/L of glucose, xylose, or arabinose as a sole carbon source. The strain was able to utilize xylose and arabinose for lipid fermentation, and assimilated those sugars in the same manner as glucose; approximately 83 % of each sugar was consumed in 10 days (Fig. 3). When *P. hubeiensis* IPM1-10 was cultivated in a medium containing 35 g/L glucose, 35 g/L xylose, or 35 g/L arabinose for 10 days, the lipid content per dry weight of cells was 21.61, 24.59 and 17.26 %, respectively (Fig. 4). The mass of accumulated lipid per cell was highest using xylose.

The fatty acid compositions of *P. hubeiensis* IPM1-10 after the 10-day culture are shown in Table 3. Although slight differences can be seen among the fatty acid compositions, the predominant fatty acids found in all cultures were palmitic (C16:0), stearic (C18:0), oleic (C18:1) and linoleic (C18:2) acids.

### Mixed sugar consumption and lipid production by *P. hubeiensis* IPM1-10

**Fig. 5:**
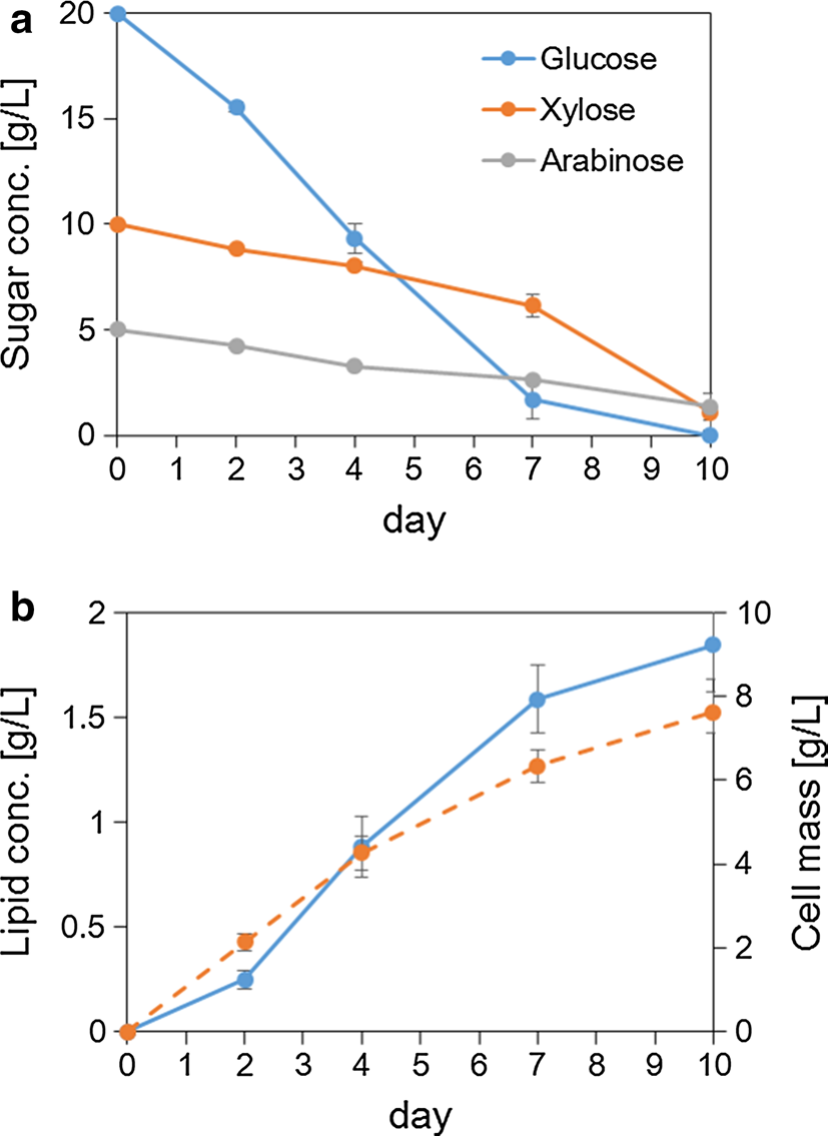
Time-course analyses of *P. hubeiensis* IPM1-10: consumption of sugars (**a**); lipid concentration (*solid line*) and cell mass (*broken line*) (**b**) in mixed-sugar medium. Data are mean ± standard deviation (*error bars*) of three assays

To demonstrate the sugar-assimilating ability of *P. hubeiensis* IPM1-10 using sugar mixtures as the carbon source, a time-course analysis of sugar consumption, lipid concentration, and cell mass was carried out (Fig. 5). The initial concentrations of glucose, xylose, and arabinose were 20, 10 and 5 g/L, respectively. The sugars decreased simultaneously rather than preferentially, although glucose was used at a higher rate (Fig. 5a). After 10 days of cultivation, 93.06 % of sugars had been consumed, which was comparable to the results of the secondary screening.

## Discussion

As shown in Table 1, the hydrolysate of lignocellulosic biomass mainly contains 3 kinds of sugars: glucose, xylose, and arabinose. The ratio of the released sugars varies, depending on the raw material types and pretreatment conditions; the ratio of hexoses to pentoses generally ranged from 1.5:1 to 3:1 (Huang et al. 2013). The final product contains a not negligible amount of arabinose. In the present study, glucose, xylose, and arabinose concentration were set at 20, 10 and 5 g/L, respectively. These concentrations are within the measured values, and were appropriate as the screening medium.

**Table 1:** Sugar composition of lignocellulosic hydrolysates

Material	Glucose [g/L]	Xylose [g/L]	Arabinose [g/L]	Total [g/L]	References
Rice straw	15.5	84.3	17.1	116.9	Huang et al. (2009)
Rice straw	22.6	79.3	13.4	115.3	Roberto et al. (1995)
Rice straw	55	10	3	68	Oberoi et al. (2012)
Rice bran	43	5	2	50	Tsigie et al. (2012)
Sugarcane bagasse	4	14	3	21	Tsigie et al. (2011)
Wheat straw	30	25	5	60	Zhang et al. (2014)
Bagasse	16.8	92.9	11.4	121.1	Huang et al. (2012)

Interestingly, all selected strains belonged to the *Ustilaginales* species (Table 2). Incidentally, IP056 is assumed to be a new species in clade 7 of Wang et al. (2015), as the sequence of the D1/D2 region of LSU rRNA gene showed a 5-nucleotide difference from that of *Macalpinomyces viridans* (HQ013125) and a 6-nucleotide difference from that of *Macalpinomyces spermophorus* (HQ013130), respectively. According to Wang et al., the species in clade 7 was not reclassified due to the taxonomic confusion of teleomorphic genera; consequently we treat this strain as an unidentified yeast strain (Wang et al. 2015). Furthermore, the selected strains (except for *Moesziomyces aphidis* RS041) were isolated from plants (leaf surface) collected on Iriomote Island. As previously reported, *Ustilaginales* species are generally distributed on the surface of leaves (Wang et al. 2006; Yoshida et al. 2014). This suggests that the inhabitants of the phyllosphere are associated with the fermentation ability of lignocellulosic sugars. Although this phenomenon is not presently understood, it is likely that the strains assimilate lignocellulose degradation products supplied by themselves or another microorganism. The xylanases-producing ability of the species has actually been reported (Adsul et al. 2009). Another feature of *Ustilaginales* species is their biosurfactant-producing ability (Jaseetha and Das 2013; Morita et al. 2010); namely, the strain can accumulate lipid intracellularly and/or produce biosurfactant extracellularly. This is the first report of mixed-sugar fermentation and of lipids accumulation using *Ustilaginales* species.

**Table 2:** Yeast species, source, and JCM number of 12 selected oleaginous yeasts

Strain	Species	Source	JCM number
IPM1-7	*Pseudozyma hubeiensis*	Plant, Iriomote Island	24583
IPM1-9	*Pseudozyma hubeiensis*	Plant, Iriomote Island	24584
IPM1-10	*Pseudozyma hubeiensis*	Plant, Iriomote Island	24585
RS041	*Moesziomyces aphidis*	Soil, Rishiri Island	24586
IP068	*Pseudozyma hubeiensis*	Plant, Iriomote Island	24587
IP045	*Pseudozyma hubeiensis*	Plant, Iriomote Island	24588
IP037	*Ustilago siamensis*	Plant, Iriomote Island	24589
IP040	*Moesziomyces antarctica*	Plant, Iriomote Island	24590
IP026	*Pseudozyma hubeiensis*	Plant, Iriomote Island	24591
IP004	*Pseudozyma hubeiensis*	Plant, Iriomote Island	24592
IP056	Unidentified *Ustilaginales* species	Plant, Iriomote Island	24593
IPM46-16	*Anthracocystis elionuri*	Plant, Iriomote Island	24544

As shown in Fig. 2, all 12 candidates showed favorable results in terms of the assimilation of pentoses. The lipid concentration of *M. aphidis* RS041, *U. siamensis* IP037, *M. antarctica* IP040, and *A. elionuri* IPM46-16 were relatively higher from the viewpoint of sugar yield (g of lipid produced per g of sugar consumed). However, their sugar consumption was not comparable to that of *P. hubeiensis* IPM1-10, which led to the lower lipid productivity (duration of time needed for lipid concentration), because the slow sugar uptake increased cultivation time. Lipid productivity is considered to be the most important parameter. Higher lipid productivity decreases production cost. In the selected strain, *P. hubeiensis* IPM1-10, the highest lipid concentration and cell mass were achieved with almost complete utilization of the sugars.

Similar to the other *Ustilaginales* species, *P. hubeiensis* has been recognized as a biosurfactant producer (Konishi et al. 2008). *P. hubeiensis* produces lipases, assimilates oil (soy oil or bovine fat), and secrets biosurfactant (Bussamara et al. 2010, 2012). Since *P. hubeiensis* can also convert lignocellulosic sugars to lipid, it has great potential for utilization of unused biomass and low-cost raw materials.

As shown in Fig. 4, the lipid-producing ability using arabinose was 30 % lower than those using glucose and xylose, even though the sugar consumption rates were similar (Fig. 3). The data suggested that arabinose was a less effective carbon source for *P. hubeiensis* IPM1-10 in terms of lipid concentration. It seems that the assimilated arabinose converted to lipid and supported cell growth at the same time, because no significant difference was observed in the cell mass between carbon sources (Fig. 4). The fatty acid composition of the lipid accumulated in *P. hubeiensis* IPM1-10 (Table 3) was similar to that of plant oil, which consists mainly of C16 and C18. These fatty acids are widely applicable, e.g., for biodiesel, chemicals, and toiletries. Compared to plant oil, lipid from oleaginous yeast is advantageous in terms of elements of economical production, such as reductions in the lifecycle, the amount of land required, and the effects of climate.

**Table 3:** Fatty acid composition of *P. hubeiensis* IPM1-10 after a 10-day culture

Carbon source	C12:0	C14:0	C16:0	C16:1	C18:0	C18:1	C18:2	C22:0	C24:0
Glucose	2.9	1.5	18.1	0.2	21.4	25.1	18.2	3.5	8.7
Xylose	0.2	1.3	22.8	0.5	16.4	26.7	21.9	3.4	6.9
Arabinose	0.9	1.3	20.4	0.3	19.8	33.6	11.3	3.3	9.1
Glucose, xylose and arabinose	2.7	1.4	19.5	0.3	20.0	26.6	17.6	3.4	8.2

Data are mean of three independent assays

When grown in the mixed-sugar medium, *P. hubeiensis* IPM1-10 required a 10-day culture. There have been several previous reports on lipid production by oleaginous yeast from mixtures of glucose, xylose, and arabinose. Sugar exhaustion was achieved at 11 days from rice straw hydrolysate by *T. fermentans* (Huang et al. 2009), 10 days from a semi-defined medium by *T. fermentans* (Huang et al. 2014), and 7 days from sugarcane bagasse hydrolysate by *Y. lipolytica* (Tsigie et al. 2011). Further consideration is needed to determine how best to improve fermentation conditions. On the other hand, to increase lipid accumulation, continuous or fed-batch culture might be effective (Gong et al. 2012; Zhao et al. 2008).

When the sugar mixtures were used as the carbon source, the lipid concentration was higher than with glucose alone. Increasing the proportion of pentoses in the carbon source increased lipid accumulation. Papanikolaou and Aggelis indicated that xylose affected lipid yield rather than glucose, because oleaginous microorganisms exclusively utilize the phosphoketolase pathway for xylose (Papanikolaou and Aggelis 2011). Therefore, *P. hubeiensis* IPM1-10 provides an efficient process for converting lignocellulosic biomass, such as the glucose, xylose, and arabinose present in hydrolysates, into lipid.

Comprehensive screening of oleaginous yeasts capable of simultaneously utilizing glucose, xylose, and l-arabinose was performed. Among the strains tested here, *P. hubeiensis* IPM1-10 had the best lipid productivity grown on lignocellulosic sugars. The strain may also be useful as a genetic resource for engineering pentoses metabolism in oleaginous microorganisms in order to improve their ability to convert sugar mixtures to lipid. More importantly, the absence of glucose repression could facilitate further study to unravel the unique sugar-assimilation mechanism.
